# Meta-Analysis of Efficacy and Safety of Karelizumab Combined with Apatinib in the Treatment of Advanced Gastric Cancer

**DOI:** 10.1155/2022/6971717

**Published:** 2022-09-22

**Authors:** Haipeng Liu, Yuanyuan Li, Yadong Yao, Kang Chen, Jianxin Gan

**Affiliations:** ^1^Department of General Surgery, Lanzhou University Second Hospital, Lanzhou, Gansu 730030, China; ^2^The Second Clinical Medical College, Lanzhou University, Lanzhou, Gansu 730030, China; ^3^Key Laboratory of Digestive System Tumors fo Gansu Province, Lanzhou University Second Hospital, Lanzhou, Gansu 730030, China

## Abstract

**Objective:**

To systematically evaluate the clinical efficacy and safety of karelizumab combined with apatinib in the treatment of advanced gastric cancer.

**Methods:**

The published databases were searched by computer, Chinese: China Biomedical Literature Database (CBM), Wanfang Journal Database, China national knowledge infrastructure (CNKI), and China Science and Technology Journal Database (VIP); English: Embase, Cochrane library, and PubMed. The search time is from the establishment of the database to May 2022, and clinical randomized controlled trials (RCT) with advanced gastric cancer as the research object and karelizumab combined with apatinib as the research variables are collected. According to the bias risk evaluation standard of Cochrane System Evaluator's Manual, the literatures meeting the inclusion standard were evaluated for bias risk, and the meta-analysis was conducted by Review Manager 5.3*. Results.* A total of 20 articles with 1150 patients were included in this study. All the included 20 articles reported objective remission rate (ORR), and the heterogeneity among 20 studies was low (*P* > 0.05, *I*^2^ = 0%). The ORR of gastric cancer patients in the observation group was significantly higher than that in the blank group [odds ratio (OR) = 1.97, 95% CI [1.53, 2.62], *P* < 0.01). All the included 20 articles reported disease control rate (DCR), and the heterogeneity among 20 studies was low (*P* = 0.87, *I*^2^ = 0%). The ORR of gastric cancer patients in the observation group was significantly higher than that in the blank group (OR = 3.09, 95% CI [2.29, 4.16], *P* < 0.01). Three articles in the included literature reported the median OS, and the heterogeneity among the three studies was low (*P* = 0.70, *I*^2^ = 0%). The median OS of gastric cancer patients in the observation group was significantly higher than that in the blank group (MD = 3.97, 95% CI [3.61, 4.39], *P* < 0.01). There are three reports on median progression-free survival (PFS) in the included literature, and there is high homogeneity among the three studies (*P* < 0.00001, *I*^2^ = 86%). There is no statistical difference between the median PFS of gastric cancer patients in the observation group and the blank group (MD = 1.21, 95% CI [−1.20, 3.70], *P* = 0.29). The incidence of hypertension in the observation group was significantly higher than that in the blank group [OR = 6.19, 95% CI (1.91, 20.20), *P* = 0.003]. The incidence of proteinuria in the observation group was significantly higher than that in the blank group [OR = 3.97, 95% CI (1.08, 14.59), *P* = 0.03]. There was no significant difference in the incidence of other adverse reactions such as hand-foot syndrome, diarrhea, and myelosuppression between the observation group and the blank group. The levels of IFN-*γ* and TNF-*α* in the observation group were significantly higher than those in the blank group (*P* < 0.0001). The levels of IL-10, IL-4, and tumor markers in the observation group were significantly lower than those in the blank group (*P* < 0.05). Egger's test showed that there was no publication bias in the 20 included studies (*P* > 0.05).

**Conclusion:**

Karelizumab combined with apatinib is effective in the treatment of advanced gastric cancer, with low incidence of adverse reactions and high safety. However, a large number of multicenter, large sample size, and high-level RCT are needed for clinical verification.

## 1. Introduction

Gastric cancer (GC) is one of the common malignant tumors in the Gastroenterology Department. Gastric cancer is not easy to be detected in the early stage, but usually it has developed to the advanced stage when it is found, with poor prognosis and high mortality, which can be ranked among the top three malignant tumors in the world [1]. At present, the clinical treatment of advanced gastric cancer is mostly chemotherapy, and the applied drugs are albumin-bound paclitaxel and docetaxel. However, with the development of the patient's condition, the resistance of conventional chemotherapy drugs is gradually improved, and the clinical application effect is significantly reduced [2]. Since very many pathological blood vessels are produced in the disease progression of advanced gastric cancer, antiangiogenic drugs such as apatinib are also commonly used in the treatment of advanced gastric cancer. Apatinib is a novel targeted therapeutic drug that can inhibit the intracellular synthesis of tranexanase by tumor vascular endothelial growth factor receptor, which can attenuate the proliferative activity of tumor blood vessels and ultimately play a role in inhibiting tumor proliferation [3, 4].

Malignant tumors are characterized by immune escape, that is, tumor cells have the characteristic of escaping from the surveillance of the immune system to achieve infinite proliferation. Programmed cell death receptor-ligand 1 (PD-L1) is an important basis for immune escape of tumor cells. PD-L1 binding to PD-1 on the surface of T cells inhibits the physiological function of T cells and helps tumor cells escape immune surveillance [5, 6]. Clinically, the T cell function is recovered by blocking the combination of PD-L1 and PD-1, which inhibits the immune escape of tumor cells and prevents further tumor deterioration. Multiple studies have shown that PD-1 inhibitors have played a good antitumor role in many kinds of malignant tumor diseases, such as primary liver cancer and esophageal squamous cell carcinoma [7, 8]. Clinically, the commonly used PD-1 inhibitor is karelizumab, which can bind to PD-1 to prevent the binding of PD-1 and PD-L1, to restore the physiological function of T cells and exert an antitumor effect [9].

There are few studies on the combination treatment of advanced gastric cancer with karelizumab and apatinib. There is no clear conclusion on the clinical efficacy and safety of the combination treatment. Meanwhile, the observation indexes of the studies conducted by different scholars are not consistent. In this paper, we systematically evaluated the clinical effectiveness and safety of the combination treatment of karelizumab and apatinib in the currently published clinical trials of the combination treatment of advanced gastric cancer (randomized controlled study, RCT), so as to provide a reference for the clinical application of the combination treatment of karelizumab and apatinib in the treatment of advanced gastric cancer.

## 2. Material and Methods

### 2.1. Criteria of Inclusion and Exclusion

#### 2.1.1. Research Object

Patients with Advanced Gastric Cancer Diagnosed by Domestic and Foreign Diagnostic Guidelines and Clinical Histopathology

#### 2.1.2. Research Method: RCT

*(1) Treatment Plan*. The blank group was treated with karelizumab; the observation group was treated with the combination of karelizumab and apatinib on the basis of the blank group.

*(2) Outcome Indicators*. Including one or more of the following indicators: median progression-free survival (PFS), disease control rate (DCR), objective remission rate (ORR), Th1, Th2, median overall survival (OS), related tumor markers, and adverse reactions.

*(3) Exclusion Criteria*. (1) The data is incomplete and cannot be completely extracted. (2) Literature belongs to the category of review, report, and conference, (3) involving animal experiments. (4) It does not meet the criteria of advanced gastric cancer. (5) Literature research methods, treatment plans, and outcome indicators are inconsistent with the requirements.

### 2.2. Literature Retrieval Strategy

#### 2.2.1. Search Databases

China national knowledge infrastructure (CNKI), Palm Bridge Research, Wanfang, China Science and Technology Journal Database (VIP), Chinese Medical Journal Full-text Database, Embase, Cochrane library, and PubMed, etc.

#### 2.2.2. Retrieval Time

From the Beginning of Database Establishment to May 2022

#### 2.2.3. Search Keywords

Gastric cancer, karelizumab, apatinib mesylate, apatinib, etc. and their corresponding English (i.e., gastric cancer, karelizumab, S-1, APA imatinib mesylate, apatinib). The keywords are combined according to the database retrieval rules.

### 2.3. Data Extraction and Quality Evaluation

Two high-quality evaluators are set up to screen, evaluate and extract data from the retrieved literature based on the inclusion criteria and exclusion criteria, and delete the literature that does not meet the criteria. The data extraction mainly includes the author, published literature year, test sample size, age, gender, basic condition, intervention measures, treatment methods, curative effect, and safety after treatment.

### 2.4. Statistical Methods

Meta-analysis of the extraction results was carried out by using Review Manager 5.3 software. Relative risk (RR) value was used for technical data, standard deviation was used for continuity variables, and the effective values of both were expressed by 95% CI value. Heterogeneity index is used to analyze the heterogeneity among the results, and *P* > 0.05 and *I*^2^ ≤ 50% indicates that the results have high homogeneity, and fixed effect model is used to analyze the data. Results of *P* ≤ 0.05 and *I*^2^ > 50%, the random effect model was used for sensitivity analysis.

## 3. Results

### 3.1. Screening Process and Results of Literature

**Figure 1:**
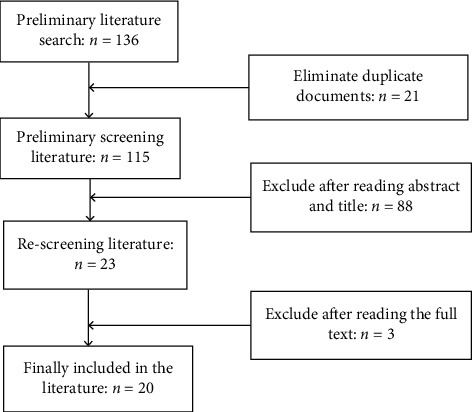
Screening process and results of literature.

A total of 136 related literatures were searched in the initial search. After the duplicate literatures were eliminated, the titles and abstracts of the literatures were read. Based on the inclusion criteria and exclusion criteria, 20 literatures were finally included in this systematic analysis [10–29]. The screening process is shown in Figure 1.

### 3.2. Basic Characteristics of Included Literature

A total of 20 articles were included in this study, with a total of 1150 patients, which was recorded in Table 1 for the basic characteristics of the literature.

### 3.3. Literature Quality

**Figure 2:**
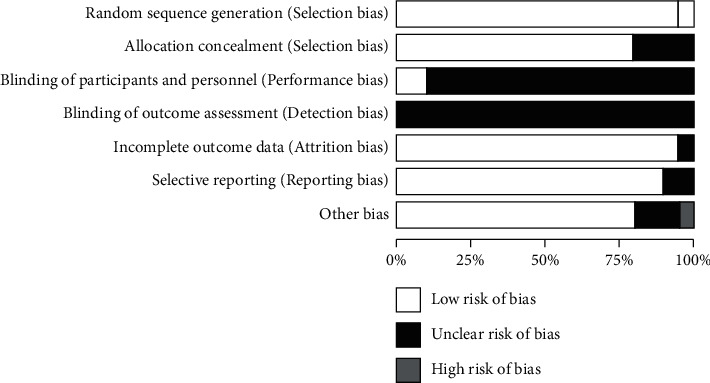
Bias analysis chart of the proportion of bias risk items in 20 included studies.

Review Manager 5.3 software was used to evaluate the literature quality of 20 included [10–29] literatures by Cochrane risk bias evaluation tool. The overall quality of the 20 included in this study was low. See Figure 2 for details.

### 3.4. Meta-Analysis Outcome

#### 3.4.1. ORR

**Figure 3:**
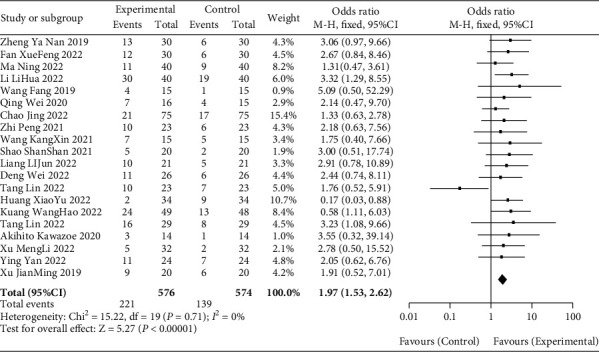
Metaforest diagram analysis of ORR of patients between observation group and blank group.

All the included 20 literatures [10–29] reported ORR, and the heterogeneity among 20 studies was low (*P* > 0.05, *I*^2^ = 0%). The fixed effect model was used to analyze the data, which showed that the ROO of gastric cancer patients in the observation group was significantly higher than that in the blank group (OR = 1.97, 95% CI [1.53, 2.62], *P* < 0.01), as recorded in Figure 3 for details.

#### 3.4.2. DCR

**Figure 4:**
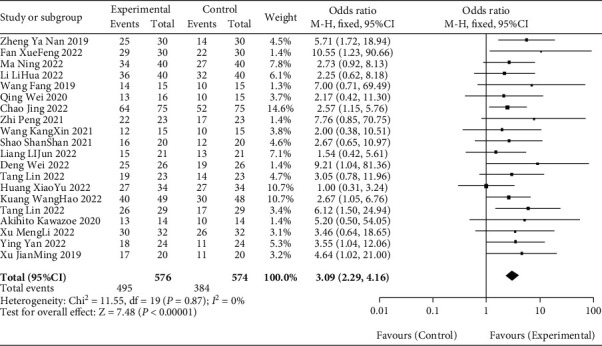
Meta-analysis forest diagram of DCR between patients in blank group and observation group.

All the included 20 literatures [10–29] reported DCR, and the heterogeneity among 20 studies was low (*P* = 0.87, *I*^2^ = 0%). The fixed effect model was used to analyze the data, which showed that the ROO of gastric cancer patients in the observation group was significantly higher than that in the blank group (OR = 3.09, 95% CI [2.29, 4.16], *P* < 0.01), as recorded in Figure 4 for details.

#### 3.4.3. Median OS

**Figure 5:**
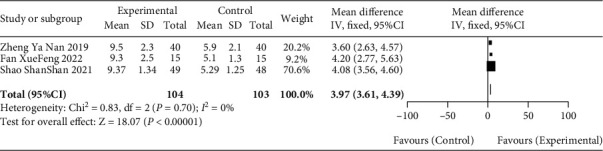
Meta-analysis forest diagram of median OS between patients in blank group and observation group.

Three papers [10, 11, 14] included in the literature reported the median OS, and the heterogeneity among the three studies was low (*P* = 0.70, *I*^2^ = 0%). Using the fixed effect model to analyze the data, the median OS of gastric cancer patients in the observation group was significantly higher than that in the blank group (MD = 3.97, 95% CI [3.61, 4.39], *P* < 0.01), as recorded in Figure 5 for details.

#### 3.4.4. Median PFS

**Figure 6:**
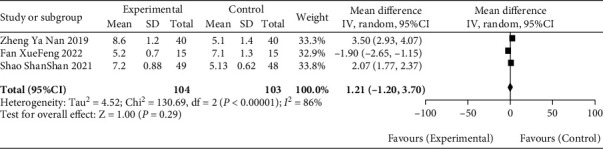
Meta-analysis forest diagram of median PFS between patients in blank group and observation group.

A median PFS was reported in three of the included articles [10, 11, 14], and there was high homogeneity between the three studies (*P* < 0.00001, *I*^2^ = 86%). Data analysis using a random-effect model showed no statistical difference between the median PFS of gastric cancer patients in the observation group and the blank control group (MD = 1.21, 95% CI [−1.20, 3.70], *P* = 0.29), as recorded in Figure 6 for details.

#### 3.4.5. Adverse Reactions

Meta-analysis results of incidence rates of adverse reactions after patient intervention in blank group and observation group. The incidence rate of hypertension in patients of observation group was significantly higher than that of blank group [OR = 6.19, 95% CI (1.91, 20.20), *P* = 0.003]. The incidence of proteinuria in patients of the observation group was significantly higher than that of the blank control group [OR = 3.97, 95% CI (1.08, 14.59), *P* = 0.03]. There was no statistically significant difference in the incidence of other adverse reactions such as hand-foot syndrome, diarrhea, and bone marrow suppression between the observation group and the blank group, as shown in Table 2 for details.

#### 3.4.6. Related Cytokines

The levels of related cytokines after treatment in patients of blank group and observation group were compared and analyzed. The results of the meta-analysis showed that the levels of IFN-*γ* and TNF-*α* in the observation group were higher than those in the blank group with significant difference (*P* < 0.0001). The levels of IL-4, IL-10, and tumor markers (CA199, TSGF, and CEA) in the observation group were significantly lower than those in the control group (*P* < 0.05). See Table 3 for details.

### 3.5. Sensitivity Analysis

In this study, the sensitivity of median PFS with greater heterogeneity was analyzed, and the analysis model was changed and the corresponding literature was screened. The final meta-analysis graph was basically consistent with the previous one, which indicates that the conclusion of the study is highly reliable.

### 3.6. Bias Test

**Figure 7:**
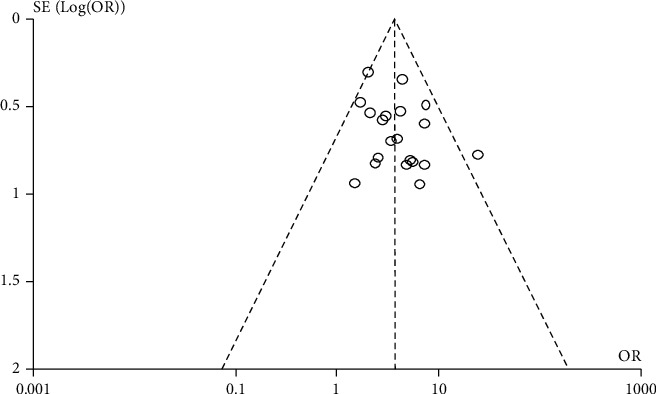
Funnel chart of clinical treatment efficiency of 20 included studies.

The inverted funnel plot was used to detect the publication bias of relevant studies with the clinical treatment efficiency as the index, and the shape of the funnel plot was relatively symmetrical. The results of Egger's test showed that there was no publication bias in the 20 included studies (*P* > 0.05). As shown in figure 7.

## 4. Discussions

As a new oral small molecule VEGFR-2 tyrosine kinase inhibitor, apatinib can effectively inhibit tumor angiogenesis to achieve the purpose of anticancer [30]. In 2014, apatinib was approved for marketing in China for the treatment of clinically advanced GC and gastroesophageal junction carcinoma (EGJC) at three-line and above. In a Phase III randomized, double-blind trial of apatinib in patients with advanced or metastatic GC and EGJC who failed second-line therapy or above, it was concluded that compared with the control group, the trial group significantly improved the median OS (6.5 months/4.7 months) and median PFS (2.6 months/1.8 months) [31]. Based on the summary of Phase I-III clinical trials, it was found that the adverse reactions of apatinib treatment included proteinuria, hypertension, hand-foot syndrome, bone marrow suppression, gastrointestinal reactions, and fatigue. The phenomenon of primary and acquired drug resistance in clinical practice also suggests that the efficacy of single-agent therapy is limited. More and more studies have proved that the combination of antiangiogenic drugs and PD-1 inhibitors may exert synergistic effects on the microenvironment of tumor-induced immunosuppression and help to improve the clinical efficacy [32, 33]. Angiogenesis and immune escape are closely related to the growth, invasion and metastasis of tumor cells. The rich blood supply characteristics and immunogenicity of gastric cancer tissues provide a scientific basis for the combination of antiangiogenesis and immune clinical treatment. Xu et al. [29] took advanced GC/EGJC as the research subject and adopted the combination therapy of karlelizumab and apatinib. After the intervention, the mPFS of the patient was 2.9 months, DCR was 78.3%, and median OS was 11.4 months. The combined application effect of the PD-1 inhibitor and the antiangiogenic agent has a higher antitumor effect than the single anti-PD-1 inhibitor therapy, which may be due to the activation of the immune checkpoint, the recovery of T cell activity, and the attack of the immune program on tumor cells [26]. At the same time, the apatinib can alleviate the hypoxia of tissues, improve the infiltration degree of CD8 + *T* cells, inhibit the generation of tumor neovascularization, and promote the restoration of normal distorted blood vessels. Remodeling the tumor microenvironment and promoting the PD-L1 expression level on the surface of tumor cells can inhibit the growth of tumor cells and exert greater antitumor effect synergistically [4, 34].

In this study, a meta-analysis was performed on published articles concerning clinical ROC trials of the combination of karlelizumab and apatinib for the treatment of advanced gastric cancer in multiple databases. Eventually, 20 articles were included in the study. The results of comparison between the combined treatment of advanced gastric cancer with karlelizumab and apatinib and monotherapy showed that the clinical treatment had a high efficiency, significantly increased the ORR, DCR, and median OS of the patients, and made the outcome indicators ORR, DCR, and median OS of the observation group significantly better than those of the blank control group. In the adverse reactions after treatment, the incidences of proteinuria and hypertension in the observation group were higher than those in the blank control group, and there was no significant difference in the incidence of the remaining adverse reactions, indicating that the combination of karlelizumab and apatinib was relatively safe. Among the secreted cytokines, the Th1 secretion level of the observation group was significantly higher than that of the control group, and the Th2 secretion level and tumor markers such as TSGF, CEA, and CA199 were significantly lower than those of the control group. Besides, sensitivity analysis indicated that the research results had high stability.

There were still certain shortcomings in this study. (1) The origin, specification, and model of the drugs used in the 20 articles were different, and the effect on the stability of the results was not known. (2) The observation indicators of the results of the 20 included studies were different, which might result in the non-disclosure of the negative results of some indicators, publication bias, and more relevant clinical trials to verify the conclusions. (3) The sample size of the 20 included articles is generally small and most of them are single-center studies, and further large sample size and multicenter clinical trials are required for conclusion verification.

In summary, the existing published clinical efficacy of the combination of karelizumab and apatinib for treating advanced gastric cancer is ideal, the clinical symptoms of patients can be remarkably improved, and the safety is high. However, the quality of included articles is generally not high, and there are large differences in intervention conditions, so blind method is not paid enough attention. This needs further optimization of relevant clinical studies and double-blind, multicenter, large sample size, and long-term follow-up study for further conclusion verification.

## Figures and Tables

**Table 1:** Basic characteristics of included literature.

Author (years)	Age (years)	Research objects	Sample size (*n*)	Intervention measures of experimental group	Outcome indicators
Zheng et al. [10]	18-75	AGC	49/48	Therapeutic effect of karelizumab + apatinib + SOX	①②③④⑤⑧⑨⑩
Fan [11],	*n*	AGC	40/40	Therapeutic effect of karelizumab + apatinib	①②③⑨⑩
Ma et al. [12]	18-75	MGC	23/23	Therapeutic effect of karelizumab + apatinib	①②③
Li et al. [13]	18-75	AGC	20/20	Therapeutic effect of karelizumab + apatinib	①②③
Wang [14],	≥18	First line and above AGC	29/29	AGC effect of karelizumab + apatinib	①②③
Wei et al. [15]	30-74	First line and above AGC	24/24	Therapeutic effect of karelizumab + apatinib 250 mg	①②③④⑥
Jing et al. [16]	18-80	First line and above AGC	40/40	Therapeutic effect of karelizumab + apatinib	①②③
Peng et al. [17]	≥18	First line and above AGC	30/30	Therapeutic effect of karelizumab + apatinib	①②③⑧
Wang et al. [18]	18-75	AGC	32/32	Therapeutic effect of karelizumab + apatinib	①②③
Shao and Xu [19]	≥18	AGC	15/15	Therapeutic effect of karelizumab + apatinib	①②③④⑦⑧⑨⑩
Liang [20]	≥18	AGC	20/20	Therapeutic effect of karelizumab + apatinib	①②③⑧
Deng et al. [21]	≥18	AGC	14/14	Therapeutic effect of karelizumab + apatinib	①②③
Tang et al. [22]	18-75	First line and above AGC	15/15	Therapeutic effect of karelizumab + apatinib	①②③
Huang et al. [23]	≥18	AGC	16/15	Therapeutic effect of karelizumab + apatinib + xaliplatin + S − 1	①②③
Kuang [24],	18-80	AGC	26/26	Therapeutic effect of karelizumab + apatinib + oxaliplatin + traditional Chinese medicine	①②③
Tang et al. [25]	≥18	First line and above AGC	75/75	Therapeutic effect of karelizumab + apatinib	①②③
Kawazoe et al. [26]	≥20	AGC	30/30	Therapeutic effect of karelizumab + apatinib	①②③
Xu et al. [27]	≥18	AGC	23/23	Therapeutic effect of karelizumab + apatinib	①②③
Yan et al. [28]	≥18	First line and above AGC	34/34	Therapeutic effect of karelizumab + apatinib	①②③
Xu et al. [29]	≥18	AGC	21/21	Therapeutic effect of karelizumab + apatinib	①②③

Note: ①: DCR: disease control rate; ②: ORR: objective remission rate; ③: related adverse reactions; ④: Th1 (IFN-*γ*, TNF-*α*); ⑤: Th2 (IL-10, IL-6); ⑥: Th2 (IL-19, IL-4); ⑦: Th2 (IL-10, IL-4); ⑧: relevant tumor markers; ⑨: median PFS: median progression-free survival; ⑩: median OS: median overall survival.

**Table 2:** Meta-analysis results of adverse reactions between patients in blank group and observation group.

Adverse reactions	Number of studies	Sample size (*n*)	OR	95% cl	*P*
Nausea or vomit	16	911	0.85	[0.61,1.16]	0.30
Hypertension	II	565	6.19	[1.91, 20.20]	0.003
Diarrhea	15	823	0.93	[0.62, 1.40]	0.72
Hand-foot syndrome	14	798	1.35	[0.92, 1.98]	0.13
Proteinuria	II	735	3.97	[1.08, 14.59]	0.03
Myelosuppression	15	878	1.08	[0.72, 1.61]	0.72
Liver damage	4	338	0.87	[0.31, 2.47]	0.79
Mucositis	6	246	1.29	[0.69, 2.44]	0.42
Weakness	13	701	1.08	[0.75,1.54]	0.69
Neuron toxication	6	396	1.15	[0.72, 1.84]	0.55
Haemorrhagia	3	276	1.52	[0.61, 3.76]	0.37
Rash	2	230	1.75	[0.52, 5.90]	0.37

**Table 3:** Meta-analysis of cytokine levels in blank group and observation group after treatment.

Cytokines	Number of studies	Sample size (*n*)	OR	95% CI	*P*
IFN-*γ*	3	175	3.22	[1.91, 4.53]	<0.00001
TNF-*α*	3	175	1.60	[1.45, 1.75]	<0.00001
IL-10	2	127	-2.79	[-4.06, -1.52]	<0.001
IL-4	2	78	-2.08	[-2.71, -1.45]	<0.00001
TSGF	2	157	-12.27	[-21.67, -2.86]	0.01
CA199	4	227	-3.89	[-4.60, -3. 19]	<0.00001
CEA	4	227	-0.06	[-0.82, -0.51]	<0.00001

## Data Availability

The labeled dataset used to support the findings of this study are available from the corresponding author upon request.
